# Addendum: Global mean nitrogen recovery efficiency in croplands can be enhanced by optimal nutrient, crop and soil management practices

**DOI:** 10.1038/s41467-025-62095-0

**Published:** 2025-08-11

**Authors:** Luncheng You, Gerard H. Ros, Yongliang Chen, Wim de Vries

**Affiliations:** 1 National Engineering Research Center for Efficient Utilization of Soil and Fertilizer Resources, College of Recourses and Environment, Shandong Agricultural University, Taian, Shandong, 271018 China; 2 College of Resources and Environmental Sciences; National Academy of Agriculture Green Development, Key Laboratory of Plant-Soil Interactions, Ministry of Education, China Agricultural University, Beijing, 100193 China; 3 Wageningen University and Research, Earth Systems and Global Change Group, P.O. Box 47, 6700AA Wageningen, the Netherlands

**Addendum to:** *Nature Communications* 10.1038/s41467-023-41504-2, published online 16 September 2023

In our recent publication titled “Global mean nitrogen recovery efficiency in croplands can be enhanced by optimal nutrient, crop and soil management practices”^1^, we presented a comprehensive analysis of the potential for improving nitrogen recovery efficiency (NUEr) in croplands worldwide through the adoption of optimized management practices. In the published version of the study, we assumed that current management practices across global croplands were entirely conventional. This assumption implied that the identified improvements from enhanced management practices would apply across all cropland areas. The approach did not consider the existing adoption of improved management practices in various regions, which may have caused an overestimation of the potential for enhancing NUEr globally.

## Estimation of the current adoption rates of improved management practices

To assess whether this assumption has significantly influenced the potential for further enhancements in NUE, we have now incorporated an estimate of the current adoption rates of improved agronomic measures. This involved creating detailed maps of current agronomic measures using methodologies outlined by Lessmann et al.^2^. The datasets utilized included Köppen-Geiger climate classification map^3^, SPAM for land use^4^, maps of nitrogen fertilizer application rates^5^, nitrogen manure production and application rates^6^, global tillage system dataset^7^, FAO databases on cropping systems^8^, crop residue retention^9^, and crop residue burning^10^. These datasets facilitated a more accurate representation of current agronomic measures and their spatial distribution.

The assessment of agronomic measures covered various practices related to (i) nutrient management, including enhanced efficiency fertilizer, combined fertilizer, organic fertilizer, fertilizer placement, timing and rate, (ii) crop management, including crop residue retention, cover cropping and crop rotation, and (iii) soil management, including zero and reduced tillage. Combined and organic fertilizers were applied only in regions where part of the manure was not yet utilized. Enhanced efficiency fertilizer and optimized fertilizer placement and timing were only applied in low-technology cropland areas, used SPAM data^4^ to identify those areas (>50% of cropland), assuming that in high-technology areas these practices are already applied. Adjusting fertilizer rates focused on areas with low nitrogen use efficiency (NUE <0.5), based on the global NUE map derived with the IMAGE model as used by Schulte-Uebbing et al.^11^. Increased crop residue retention was assumed applicable in regions where burning of crop residues was common (>25% cropland), while cover cropping was applied in areas where in less than 25% of the area catch crops were used. Cover cropping was applied in regions (grid cells) with a cropping intensity of less than one harvest per year, as defined in the GCI (Global Cropping Intensity) dataset^12^, indicating fallow or partially cropped areas. Both zero and reduced tillage (conservation agriculture) were applied in regions with high or intermediate tillage (<50% cropland).

## Revised estimation of potential increase in nitrogen recovery efficiency by improved management

Considering the areas where it is likely that improved measures are already applied, the potential for further improvement in NUEr is substantially lower than initially estimated. This impact on the results is reflected in the updated maps presented in this Addendum as Figs. 1, 2, replacing the original Figs. 4 and 5 in ref. ^1^. Figure 1 presents the spatial variation in the mean, lower (2.5%) and upper (97.5%) boundary of absolute average NUEr changes for the combined effects of optimal nutrient management, optimal crop management and optimal soil management, while Fig. 2 does so for the mean separate effects. Where the initial estimate was a mean global potential NUE increase near 30% varying from ca. 24 to 36% (Fig. 4 in ref. ^1^) it is now near 19% varying from ca. 8 to 30% (Fig. 1). While the impact of soil and crop management stayed close to the original values near 6% and 1%, respectively, the mean impact of nutrient management reduced from ~27% (Fig. 5 in ref. ^1^) to ~17% (Fig. 2). While the initial findings underscored the significant potential for improvement, the updated analysis offers a more refined understanding of where and how much improvement can realistically be achieved, being about 35% lower than originally estimated. This update underscores the importance of considering existing practices in policy and management recommendations aimed at improving NUEr in global croplands.

**Fig. 1:**
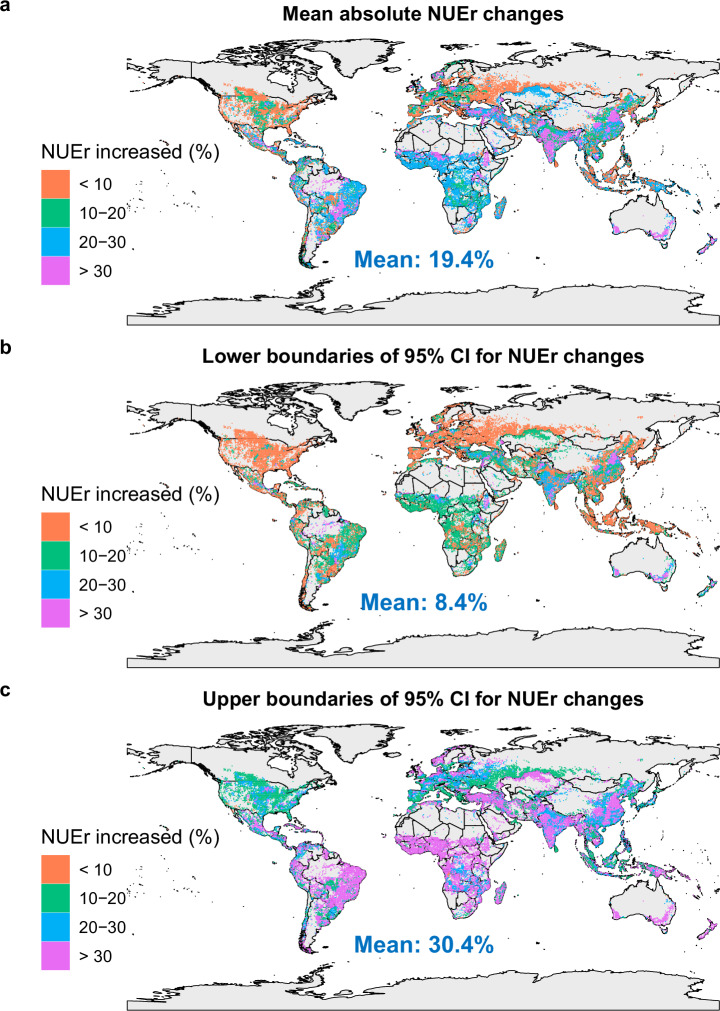
Updated original Fig. 4. Predicted spatial variation in impacts of combined optimal management practices on absolute average N recovery efficiency (NUEr) changes (%) in global croplands based on the MD (the raw mean difference) model. Source data are available alongside this amendment.

**Fig. 2:**
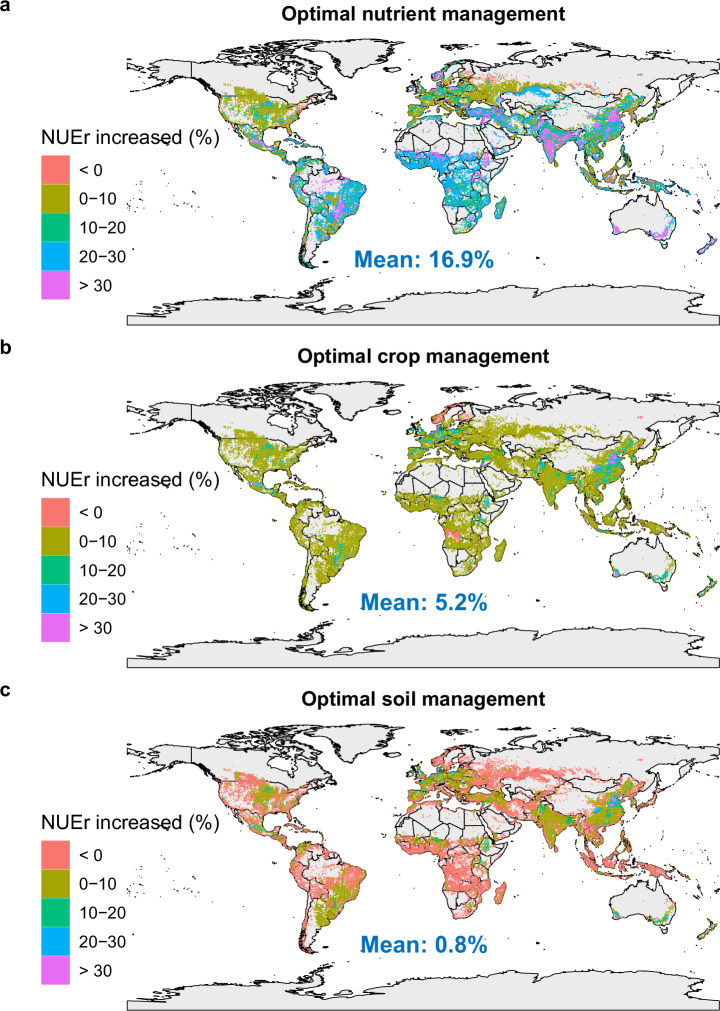
Updated original Fig. 5. Predicted spatial variation in impacts of management practices on absolute average N recovery efficiency (NUEr) changes (%) in global croplands based on the MD (the raw mean difference) model. Source data are available alongside this amendment.

## Data limitations and areas for further improvement

In our revised estimate, we assumed that the application of enhanced efficiency fertilizers (e.g., coated fertilizers or inhibitors) and optimized fertilizer placement has already been implemented in high-technology regions, while conventional fertilizers are still used in low-technology areas, while using the SPAM dataset to derive information on cropland areas with low, intermediate, and hightechnology levels. However, this assumption may not fully align with the current market realities, as enhanced efficiency fertilizers remain niche products, primarily used in special crops in North America, China, and Europe. The limited global adoption and regional disparities in fertilizer use highlight the need for more precise data on the adoption rates of these technologies. Future studies should incorporate region-specific data to improve estimates of the current use of enhanced efficiency fertilizers.

For crop residue management, we relied on the FAO database to identify regions where residue retention could be most effective, particularly in areas with significant crop residue burning (>25% of cropland). Similarly, crop rotation and cover cropping practices were assessed using global datasets on cropping intensity and catch crop potential. While these assumptions provide a useful framework, they may not fully capture regional variations or evolving local practices, potentially underestimating the dynamic nature of crop management strategies.

Soil management practices, such as zero and reduced tillage, were analyzed using a global tillage system dataset. This enabled the identification of regions with high or intermediate tillage intensity where conservation agriculture could be applied. However, the dataset may not reflect recent advancements or policy-driven shifts toward no-tillage farming in certain regions. Additionally, areas with missing data (less than 1% of total cropland) were assumed to have no adoption of these practices, which could introduce bias.

In summary, while the datasets used provided valuable insights into the adoption of either conventional or improved farming practices, they have clear limitations. Global datasets often lack the granularity to capture region-specific practices, such as local fertilizer types, cropping patterns, and tillage methods. Broad classifications, such as SPAM’s technology levels, may also overlook fine-scale heterogeneity in agricultural practices. To enhance future analyses, incorporating higher-resolution, region-specific data and up-to-date information on fertilizer adoption rates would be essential. Addressing these limitations would help refine estimates and provide a more accurate assessment of the global potential for improving nitrogen recovery efficiency.

## Description of updates to the original article

We have added the following clarification about our underlying assumptions to the Methods subsection “Assessing spatial variation and global potential to increase NUEr and its uncertainties”:

“In the analysis, we assumed that management practices across global croplands are entirely conventional, without explicitly accounting for the existing adoption of improved agronomic measures in various regions. This assumption may have led to an overestimation of the potential for enhancing nitrogen recovery efficiency (NUEr) globally. More specifically, we did not account for several aspects that have now been considered in an updated analysis published in an Addendum to this Article: (1) Regional variability in management practices. The analysis did not incorporate detailed maps or datasets reflecting the current adoption rates of improved practices, such as enhanced efficiency fertilizers, optimized fertilizer placement, cover cropping, and conservation tillage. (2) Technology-level differences. We used the SPAM dataset to classify cropland into low, high, and intermediate technology levels, but did not link implementation of conventional versus enhanced practices to the technology level as we did in the revised estimate. In the Addendum, we now also admit the limitation of the assumption that enhanced efficiency fertilizers are applied in high technology areas. (3) Crop residue and tillage practices. The analysis did not account for regional variations in crop residue management (e.g., retention vs. burning) or the adoption of conservation tillage practices. (4) Fertilizer use efficiency. Adjustments to fertilizer rates were not explicitly linked to regional differences in nitrogen use efficiency (NUE), particularly in areas with low NUE (<0.5).”

We have added the following notes to the legends of Figures 4 and 5, respectively: “For an updated version of this figure taking into account the existing adoption of combined improved management practices in various regions, please see Figure 1 in our Addendum to this Article.” and “For an updated version of this figure taking into account the existing adoption of three individual improved management practices (nutrient, crop, and soil management) in various regions, please see Figure 2 in our Addendum to this Article.”

## Source data

Source Data Figs. 1, 2
