# Supplementary material for: Global mean nitrogen recovery efficiency in croplands can be enhanced by optimal nutrient, crop and soil management practices
Source: Nat Commun. 2023 Sep 16;14:5747. doi: 10.1038/s41467-023-41504-2 (PMC10505174; doi:10.1038/s41467-023-41504-2)
Supplement: Supplementary file 1 — Supplementary Information [file 41467_2023_41504_MOESM1_ESM.pdf]

Supplementary Information

Global mean nitrogen recovery efficiency in croplands can be enhanced by optimal nutrient, crop and soil management practices

Luncheng You[1], Gerard H. Ros[2], Yongliang Chen[1,*], Qi Shao[1], Madaline D. Young[2], Fusuo Zhang[1] & Wim de Vries[2]

[1]College of Resources and Environmental Sciences, National Academy of Agriculture Green Development, Key Laboratory of Plant–Soil Interactions, Ministry of Education, State Key Laboratory of Nutrient Use and Management, China Agricultural University, 100193, Beijing, China

[2]Wageningen University and Research, Environmental Systems Analysis Group, P.O. Box 47, 6700AA Wageningen, the Netherland

*Corresponding author: Yongliang Chen.

E-mail: ylchen@cau.edu.cn.

Contents

Supplementary Note 1 The detailed search terms on the Web of Science database.

Supplementary tables:

Supplementary Table 1 Summary of 29 meta-analytical publications combined for further analysis in this study.

Supplementary Table 2 Description of the nutrient, crop and soil management variables used in the meta-model of existing meta-analytical regression models and the meta-regression of the underlying primary studies.

Supplementary Table 3 Overview of data collected of the primary studies.

Supplementary Table 4 Summary statistics of the nitrogen recovery efficiency in control and treated plots, the N dose, soil properties and climate variables in the investigated sites of the primary studies.

Supplementary Table 5 Relative and absolute changes in N recovery efficiency (NUEr) in response to management practices (meta-analytical data vs. primary data) relative to a reference practice.

Supplementary Table 6 Main factor analysis of the contribution of management practices and site conditions on the variation in N recovery efficiency (NUEr) based on the primary studies, as derived by three models (ROM, MD and SMD methods).

Supplementary Table 7 Parameter estimates for the contribution of management practices and site conditions on the variation in NUEr based on the primary studies derived by three models (ROM, MD and SMD methods) with corresponding standard errors, *p*-values and 95% confidence intervals (CI).

Supplementary figures:

Supplementary Fig. 1 PRISMA Flow diagram showing the selection process of studies included in the analysis.

Supplementary Fig. 2 Frequency distribution histogram of MAT (a), MAP (b), clay (c), SOC (d), pH (e), and N rate (f).

Supplementary Fig. 3 Data fitting and correlation coefficients among MAT, MAP, clay, SOC, pH, N rate and crop type.

Supplementary Fig. 4 Mean effects of nutrient (a), crop (b) and soil (c) management practices, based on original source data of meta-analyses (circles), on the relative change in N recovery efficiency (%) as affected by site conditions in comparison to the overall mean impact (solid line).

Supplementary Fig. 5 Upscaled results for global impacts of management practices on changes

in absolute N recovery efficiency (%) based on the MD model.

Supplementary Note 1 The detailed search terms that were used on the Web of Science database were:

TS = ((rotation cropping OR crop rotation OR crop diversification OR double cropping OR intercropping OR multi-cropping OR cover cropping OR cover crop OR catch cropping OR legume in rotation OR crop residues OR reduced tillage OR minimal tillage OR strip till OR zone till OR ridge till OR no tillage OR organic fertilizer OR manure OR combined fertilizer OR fertilizer rate OR fertilizer timing OR fertilizer placement OR enhanced efficiency fertilizer OR biofertilizer) AND (nitrogen use efficiency OR NUE OR N uptake OR crop N content) AND meta-analysis)

Supplementary Table 1 Summary of 29 meta-analytical publications combined for further analysis in this study.

			Nutrient management						Crop management			Soil management		
**Study**	**Geographic coverage**	**Crop type**	**Enhanced efficiency fertilizer**	**Combined fertilizer**	**Organic fertilizer**	**Right fertilizer placement**	**Right fertilizer rate**	**Right fertilizer timing**	**Residue retention**	**Cover cropping**	**Crop rotation**	**Zero tillage**	**Reduced tillage**	**Response variable**
Abalos et al. 2014	Global	maize, wheat, rice	√											N uptake, NUEr
Burzaco et al. 2014	Global	maize	√											N uptake, NUEr
Chivenge et al. 2011	Sub-Saharan Africa	maize		√	√									NUEr
Ding et al. 2018	China	rice	√		√				√					NUEr
Fernandez et al 2020	Global	wheat						√						NUEr
Gardner & Drinkwater 2009	Global	maize, wheat	√		√	√	√	√			√			N uptake, NUEr
Jiang et al. 2021	Global	rice	√											NUEr
Li et al. 2019	Global	maize					√							NUEr
Liang et al. 2016	Global	rice										√		N uptake, NUEr
Linquist et al. 2013	Global	rice	√											N uptake
Liu et al. 2020	China	wheat							√			√		N uptake
Liu et al. 2021a	China	maize							√					NUEr
Liu et al. 2021b	China	maize, wheat, rice			√									N uptake, NUEr
Nkebiwe et al. 2016	Global	maize, wheat, rice				√								N uptake
Qiao et al. 2015	Global	maize, wheat, rice	√											N uptake, NUEr
Quan et al. 2021	Global	maize	√	√	√	√	√	√		√		√		N uptake, NUEr
Quemada et al. 2013	Global	maize, wheat, rice	√				√	√		√				N uptake
Sha et al. 2020	Global	maize, wheat, rice	√											N uptake, NUEr
Wang et al. 2018	Global	maize, wheat, rice							√					N uptake, NUEr

			Nutrient management						Crop management		Soil management		
**Study**	**Geographic coverage**	**Crop type**	**Enhanced efficiency fertilizer**	**Combined fertilizer**	**Organic fertilizer**	**Right fertilizer placement**	**Right fertilizer rate**	**Right fertilizer timing**	**Residue retention**	**Cover cropping**	**Crop rotation**	**Zero tillage**	**Reduced tillage**	**Response variable**
Wei et al. 2020	Global	maize		√	√									NUEr
Xia et al. 2017a	China	maize, wheat, rice	√			√	√	√						N uptake, NUEr
Xia et al. 2017b	Global	wheat			√									N uptake
Xia et al. 2018	Global	maize, wheat, rice							√					N uptake, NUEr
Yang et al. 2016	Global	wheat	√											N uptake
Zavattaro et al. 2015	Europe	maize, wheat, rice			√				√	√	√	√	√	N uptake, NUEr
Zavattaro et al. 2017	Europe	maize, wheat, rice		√	√									N uptake, NUEr
Zhang et al. 2019	Global	maize	√											NUEr
Zhang et al. 2020	China	maize, wheat, rice		√	√									NUEr
Zhu et al. 2020	Global	maize, wheat, rice	√											N uptake, NUEr

Supplementary Table 2 Description of the nutrient, crop and soil management variables used in the meta-model of existing meta-analytical regression models and the meta-regression of the underlying primary studies.

**Management practice name (abbreviation)**		**Treatment description**	**Control**
Nutrient management	Enhanced efficiency fertilizer (EE)	application of enhanced efficiency fertilizers such as controlled release fertilizer, nitrification inhibitor/urease inhibitor fertilizer	not applied
	Combined fertilizer (CF)	combined organic and mineral fertilizer	mineral fertilizer
	Organic fertilizer (OF)	organic fertilizer, namely from animal waste or compost	mineral fertilizer
	Fertilizer placement (RFP)	improved/optimized placement of fertilizer	standard placement
	Fertilizer rate (RFR)	improved/optimized or reduced fertilizer rate	standard rate
	Fertilizer timing (RFT)	improved/optimized timing of fertilizer application	standard timing
Crop management	Residue retention (RES)	retaining or incorporating crop residues after harvest, mulching	removing
	Cover cropping (CC)	cover cropping, catch cropping	no cover crop
	Crop rotation (ROT)	two or more than two crops in rotation	monoculture
Soil management	Zero tillage (ZT)	no tillage	multiple-pass tillage
	Reduced tillage (RT)	reduced or minimal tillage practices such as strip till, zone till ridge till	multiple-pass tillage

Supplementary Table 3 Overview of data collected of the primary studies.

Group	Variable	Unit	Description
study	ID	-	unique ID
	reference	-	paper reference: firstauthor_year_firstwordoftitle
location	lat	-	if present: x coordinate of site
	lon	-	if present: y coordinate of site
climate	MAT	ºC	mean annual temperature
	MAP	mm	mean annual precipitation
site properties	SOC	g/kg	soil organic carbon content
	pH	-	soil acidity
	clay	%	clay content
management	crop_type	-	crop
	tillage	-	tillage system
	N_dose	kg N/ha	total N input (fertilizer and manure) in kg N/ha
	replication	-	number of replicates
response variable NUEr treatment	nuet_type	-	REN: recovery efficiency of N (kg (N Grain) kg / (N fertilized));
	nuet_mean	%	mean NUEr of treatments
	nuet_sd	-	standard deviation of NUEr of treatments
response variable NUEr control	nuec_type	-	REN: recovery efficiency of N (kg (N Grain) kg / (N fertilized));
	nuec_mean	%	mean NUEr of control
	nuec_sd	-	standard deviation of NUEr of control
response variable N uptake treatment	nut_mean	kg·N/ha	mean nitrogen uptake of treatment
	nut_sd	-	standard deviation of N uptake of treatment
response variable N uptake control	nuc_mean	kg·N/ha	mean nitrogen uptake of control
	nuc_sd	-	standard deviation of N uptake of control

Supplementary Table 4 Summary statistics of the nitrogen recovery efficiency in control and treated plots, the N dose, soil properties and climate variables in the investigated sites of the primary studies. For each variable, mean, standard deviation, minimum and maximum values are given.

**Variable**	**Unit**	**Mean**	**Standard deviation**	**Min**	**Max**
**NUEr control**	%	33	15	2	96
**NUEr treatment**	%	39	15	4.2	98
**N rate**	kg ha^{-1}	173	65	20	471
**Clay**	%	28	6.5	8.8	53
**Soil pH**	-	7	0.9	4.5	8.5
**SOC**	g kg^{-1}	14	9.3	2.7	80
**MAP**	mm	963	446	45	2330
**MAT**	°C	14	5	-0.6	29

NUEr, nitrogen recovery efficiency; N rate, N application rate; Clay, soil clay content; SOC, soil organic carbon; MAP, mean annual precipitation; and MAT, mean annual temperate.

Supplementary Table 5 Relative and absolute changes in N recovery efficiency (NUEr) in response to management practices (meta-analytical data vs. primary data) relative to a reference practice (see Supplementary Table 2 for the control/reference situation for each practice).

**Management practice**		**Relative change in NUEr (%) [mean (*95% CI*)]**		**Absolute change in NUEr (%) [mean (*95% CI*)]**		
		**Meta-analytical data**	**Primary data**	**Primary data**		
		ROM method		*ROM method*	*MD method*	*SMD method*
**Nutrient management**	EE	14 (-14, 57)	**31 (27, 34)**	**9.8 (8.6, 11)**	**7.9 (6.9, 9)**	**6.6 (5.7, 7.4)**
	CF	1.9 (-27, 33)	**16 (10, 23)**	**5.9 (3.7, 8.1)**	**4.9 (3, 6.8)**	**3.6 (2.4, 4.8)**
	OF	-1.1 (-43, 71)	-1.2 (-16, 14)	-0.5 (-6.1, 5.2)	-0.9 (-4.7, 3)	0.5 (-2.7, 3.7)
	RFP	**28 (25, 31)**	**26 (17, 34)**	**7.3 (4.8, 9.8)**	**6.6 (3.8, 9.4)**	**5 (3.2, 6.9)**
	RFR	17 (-18, 83)	**39 (34, 45)**	**11 (9.9, 13)**	**9.6 (8, 11.3)**	**7.6 (6.5, 8.8)**
	RFT	6.7 (-18, 42)	**24 (17, 31)**	**7 (4.9, 9.1)**	**6.3 (4, 8.6)**	**4.6 (3.3, 5.8)**
**Crop management**	RES	6.9 (-1, 36)	**24 (17, 32)**	**8 (5.6, 10)**	**5.9 (3.7, 8)**	**4.4 (2.8, 5.9)**
	CC	-13 (-36, 19)	**22 (6.6, 38)**	**8 (2.4, 14)**	**5 (0.6, 9.5)**	**4.9 (1.3, 8.6)**
	ROT	**38 (38, 38)**	16 (-10, 43)	4 (-2.4, 10)	2.8 (-3.5, 9.1)	3.2 (-0.7, 7.1)
**Soil management**	ZT	-2.3 (-17, 30)	-4.1 (-12, 4.3)	-1.6 (-4.8, 1.7)	-0.9 (-3.6, 1.8)	-0.7 (-2.7, 1.2)
	RT	**-9 (-9, -9)**	2.2 (-29, 34)	0.8 (-11, 12.8)	0.9 (-6.9, 8.6)	-0.1 (-5.5, 5.4)

EE: Enhanced efficiency fertilizer; CF: Combined fertilizer; OF: Organic fertilizer; RFP: Right fertilizer placement; RFR: Right fertilizer rate; RFT: Right fertilizer timing; BC: Biochar; RES: Residue retention; CC: Cover cropping; ROT: Crop rotation; RT: Reduced tillage; ZT: Zero tillage. ROM, the log transformed ratio of means (Eq. 5); MD, the raw mean difference (Eq. 8); SMD, the standardized mean difference (Eq. 10).

Supplementary Table 6 Main factor analysis of the contribution of management practices and site conditions on the variation in N recovery efficiency (NUEr) based on the primary studies, as derived by three models (ROM, MD and SMD methods): CI stands for 95% confidence intervals. Effect of moderators were tested using ANOVA using linear contrasts between model coefficients as implemented by metafor R package. Confidence intervals were derived via (non-parametric) bootstrapping as implemented by metafor R package.

**ROM**						
**Moderator**	**Moderator name**	**Parameter estimate**	**Standard error**	**CI lower boundary**	**CI upper boundary**	***p* value**
fertilizer_type	enhanced	0.397	0.015	0.368	0.427	0
fertilizer_type	combined	0.195	0.017	0.161	0.228	0
fertilizer_type	organic	0.168	0.018	0.133	0.202	0
fertilizer_type	mineral	0.09	0.015	0.061	0.119	0
fertilizer_strategy	placement	-0.03	0.017	-0.063	0.003	0.074
fertilizer_strategy	rate	0.331	0.016	0.3	0.362	0
fertilizer_strategy	timing	0.403	0.016	0.371	0.436	0
fertilizer_strategy	standard	0.185	0.014	0.158	0.213	0
crop_residue	no	0.221	0.014	0.194	0.248	0
crop_residue	yes	0.232	0.017	0.199	0.266	0
cover_crop_and_crop_rotation	no	0.219	0.014	0.192	0.245	0
cover_crop_and_crop_rotation	yes	0.305	0.028	0.251	0.359	0
tillage	zero	0.052	0.02	0.014	0.091	0.007
tillage	reduced	0.159	0.028	0.104	0.214	0
tillage	standard	0.232	0.013	0.206	0.258	0
crop_type	wheat	0.254	0.014	0.226	0.282	0
crop_type	maize	0.256	0.015	0.226	0.286	0
crop_type	rice	0.164	0.014	0.136	0.192	0
N_dose_scaled	intrcpt	0.226	0.015	0.197	0.255	0
N_dose_scaled	varsel	-0.131	0.001	-0.134	-0.129	0
clay_scaled	intrcpt	0.222	0.013	0.196	0.249	0
clay_scaled	varsel	-0.033	0.009	-0.05	-0.016	0
pH_scaled	intrcpt	0.222	0.013	0.196	0.249	0
pH_scaled	varsel	0.011	0.009	-0.006	0.028	0.199
SOC_scaled	intrcpt	0.222	0.014	0.195	0.248	0
SOC_scaled	varsel	0.012	0.007	-0.002	0.025	0.083
MAP_scaled	intrcpt	0.222	0.013	0.196	0.249	0
MAP_scaled	varsel	0.004	0.011	-0.017	0.026	0.688
MAT_scaled	intrcpt	0.222	0.013	0.195	0.248	0
MAT_scaled	varsel	-0.011	0.011	-0.034	0.011	0.314
**MD**						

**Moderator**	**Moderator name**	**Parameter estimate**	**Standard error**	**CI lower boundary**	**CI upper boundary**	***p* value**
fertilizer_type	enhanced	9.076	0.422	8.25	9.902	0
fertilizer_type	combined	7.005	0.488	6.049	7.961	0
fertilizer_type	organic	3.539	0.529	2.501	4.577	0
fertilizer_type	mineral	4.475	0.418	3.656	5.294	0
fertilizer_strategy	placement	3.441	0.473	2.515	4.368	0
fertilizer_strategy	rate	9.924	0.474	8.995	10.854	0
fertilizer_strategy	timing	8.366	0.471	7.443	9.289	0
fertilizer_strategy	standard	5.38	0.408	4.58	6.18	0
crop_residue	no	6.41	0.408	5.611	7.21	0
crop_residue	yes	6.908	0.502	5.925	7.891	0
cover_crop_and_crop_rotation	no	6.388	0.409	5.587	7.189	0
cover_crop_and_crop_rotation	yes	8.138	0.645	6.874	9.401	0
tillage	zero	2.091	0.582	0.951	3.231	0
tillage	reduced	4.969	0.97	3.069	6.87	0
tillage	standard	6.722	0.401	5.937	7.507	0
crop_type	wheat	8.55	0.428	7.711	9.39	0
crop_type	maize	6.659	0.457	5.763	7.555	0
crop_type	rice	4.643	0.429	3.803	5.483	0
N_dose_scaled	intrcpt	6.812	0.451	5.927	7.697	0
N_dose_scaled	varsel	-4.711	0.043	-4.795	-4.627	0
clay_scaled	intrcpt	6.47	0.408	5.671	7.27	0
clay_scaled	varsel	-1.424	0.258	-1.93	-0.918	0
pH_scaled	intrcpt	6.461	0.406	5.665	7.257	0
pH_scaled	varsel	0.1	0.231	-0.353	0.553	0.666
SOC_scaled	intrcpt	6.436	0.407	5.638	7.233	0
SOC_scaled	varsel	0.747	0.215	0.325	1.168	0.001
MAP_scaled	intrcpt	6.461	0.407	5.664	7.258	0
MAP_scaled	varsel	0.064	0.251	-0.428	0.556	0.8
MAT_scaled	intrcpt	6.453	0.406	5.657	7.248	0
MAT_scaled	varsel	-0.237	0.328	-0.879	0.405	0.47
**SMD**						
**Moderator**	**Moderator name**	**Parameter estimate**	**Standard error**	**CI lower boundary**	**CI upper boundary**	***p* value**
fertilizer_type	enhanced	1.369	0.076	1.22	1.518	0
fertilizer_type	combined	1.195	0.105	0.988	1.401	0
fertilizer_type	organic	0.455	0.144	0.173	0.736	0.002
fertilizer_type	mineral	0.925	0.071	0.786	1.064	0
fertilizer_strategy	placement	1.404	0.133	1.144	1.665	0
fertilizer_strategy	rate	1.57	0.089	1.396	1.744	0

fertilizer_strategy	timing	1.154	0.113	0.932	1.376	0
fertilizer_strategy	standard	0.92	0.063	0.796	1.044	0
crop_residue	no	1.102	0.062	0.981	1.223	0
crop_residue	yes	1.119	0.115	0.894	1.343	0
cover_crop_and_crop_rotation	no	1.097	0.061	0.977	1.217	0
cover_crop_and_crop_rotation	yes	1.261	0.204	0.861	1.662	0
tillage	zero	0.105	0.135	-0.159	0.369	0.437
tillage	reduced	0.654	0.195	0.272	1.035	0.001
tillage	standard	1.164	0.06	1.047	1.28	0
crop_type	wheat	1.048	0.081	0.89	1.205	0
crop_type	maize	1.111	0.083	0.948	1.273	0
crop_type	rice	1.141	0.085	0.973	1.308	0
N_dose_scaled	intrcpt	1.105	0.061	0.986	1.224	0
N_dose_scaled	varsel	-0.019	0.035	-0.088	0.05	0.588
clay_scaled	intrcpt	1.105	0.061	0.986	1.224	0
clay_scaled	varsel	-0.1	0.052	-0.201	0.002	0.054
pH_scaled	intrcpt	1.104	0.061	0.985	1.223	0
pH_scaled	varsel	0.044	0.051	-0.057	0.145	0.39
SOC_scaled	intrcpt	1.103	0.061	0.984	1.223	0
SOC_scaled	varsel	0.008	0.045	-0.081	0.097	0.86
MAP_scaled	intrcpt	1.104	0.061	0.985	1.222	0
MAP_scaled	varsel	-0.016	0.057	-0.126	0.095	0.781
MAT_scaled	intrcpt	1.102	0.061	0.983	1.22	0
MAT_scaled	varsel	-0.061	0.056	-0.171	0.049	0.28

Scaled variables were converted to have unit variance. ROM, the log transformed ratio of means (Eq. 5); MD, the raw mean difference (Eq. 8); SMD, the standardized mean difference (Eq. 10).

Supplementary Table 7 Parameter estimates for the contribution of management practices and site conditions on the variation in NUEr based on the primary studies derived by three models (ROM, MD and SMD methods) with corresponding standard errors, *p*-values and 95% confidence intervals (CI). Asterisks indicate interactions. Effect of moderators were tested using ANOVA using linear contrasts between model coefficients as implemented by metafor R package. Confidence intervals were derived via (non-parametric) bootstrapping as implemented by metafor R package.

**ROM**					**Q_E: 80253; Pseudo-R^2: 0.57; p value < 0.05**
Moderator	Parameter estimate	Standard error	*p*-value	CI lower boundary	CI upper boundary
fertilizer_type_enhanced	0.3495	0.0190	<.0001	0.3122	0.3867
fertilizer_type_combined	0.2129	0.0206	<.0001	0.1725	0.2532
fertilizer_type_organic	0.1771	0.0210	<.0001	0.1359	0.2182
fertilizer_type_mineral	0.0956	0.0195	<.0001	0.0574	0.1338
fertilizer_strategy_placement	0.1419	0.0182	<.0001	0.1063	0.1774
fertilizer_strategy_rate	0.1827	0.0115	<.0001	0.1602	0.2053
fertilizer_strategy_timing	0.2036	0.0137	<.0001	0.1768	0.2304
crop_residue_yes	0.0725	0.0127	<.0001	0.0477	0.0973
cover_crop_and/or_crop_rotation	0.1317	0.0265	<.0001	0.0798	0.1836
Tillage_zero/reduced till	-0.0638	0.0144	<.0001	-0.0920	-0.0357
crop_type_wheat	-0.0353	0.0016	<.0001	-0.0385	-0.0321
crop_type_maize	-0.0049	0.0115	0.6676	-0.0274	0.0175
crop_type_rice	0.0000	0.0000	0.0000	0.0000	0.0000
N_scaled	-1.1786	0.0088	<.0001	-1.1957	-1.1614
Clay_scaled	-0.0706	0.0115	<.0001	-0.0930	-0.0481
SOC_scaled	0.0626	0.0113	<.0001	0.0406	0.0847
pH_scaled	0.0522	0.0142	<.0001	0.0242	0.0801
MAP_scaled	0.1259	0.0191	<.0001	0.0884	0.1634
MAT_scaled	-0.0723	0.0176	<.0001	-0.1069	-0.0378
N_squared_scaled	1.1200	0.0090	<.0001	1.1024	1.1377
Placement:maize	-0.3404	0.0211	<.0001	-0.3818	-0.2991
MAT_scaled:maize	0.1322	0.0231	<.0001	0.0869	0.1775
N_scaled:SOC_scaled	0.0627	0.0040	<.0001	0.0548	0.0706
**MD**					**Q_E: 63270; Pseudo-R^2: 0.65; p value < 0.05**
Moderator	Parameter estimate	Standard error	*p*-value	CI lower boundary	CI upper boundary
fertilizer_type_enhanced	8.0954	0.5367	<.0001	7.0434	9.1473
fertilizer_type_combined	7.5767	0.5854	<.0001	6.4293	8.7242
fertilizer_type_organic	3.7867	0.6204	<.0001	2.5707	5.0027
fertilizer_type_mineral	4.8150	0.5415	<.0001	3.7536	5.8763
fertilizer_strategy_placement	1.8479	0.5348	<.0001	0.7998	2.8960
fertilizer_strategy_rate	4.3411	0.3541	<.0001	3.6470	5.0351
fertilizer_strategy_timing	3.2728	0.3493	<.0001	2.5882	3.9573
crop_residue_yes	1.8930	0.3415	<.0001	1.2237	2.5623

cover_crop_and/or_crop_rotation	1.5543	0.5517	<.01	0.4730	2.6355
Tillage_zero/reduced till	-2.8625	0.4299	<.0001	-3.7050	-2.0199
crop_type_wheat	0.1205	0.0633	0.0570	-0.0036	0.2445
crop_type_maize	-0.7213	0.3158	<.05	-1.3403	-0.1023
crop_type_rice	0.0000	0.0000	0.0000	0.0000	0.0000
N_scaled	-30.1474	0.2784	<.0001	-30.6930	-29.6018
Clay_scaled	-2.2753	0.3245	<.0001	-2.9113	-1.6394
SOC_scaled	1.1541	0.3561	<.005	0.4561	1.8521
pH_scaled	0.2732	0.3074	0.3742	-0.3293	0.8757
MAP_scaled	0.9656	0.3854	<.05	0.2103	1.7209
MAT_scaled	-0.4163	0.4925	0.3979	-1.3817	0.5490
N_squared_scaled	27.9200	0.2822	<.0001	27.3670	28.4731
Placement:maize	-3.0466	0.5610	<.0001	-4.1461	-1.9471
MAT_scaled:maize	2.3939	0.6452	<.001	1.1294	3.6584
N_scaled:SOC_scaled	0.4221	0.1229	<.001	0.1812	0.6631
**SMD**					**Q_E: 6108; Pseudo-R^2: 0.63; *p* value < 0.05**
Moderator	Parameter estimate	Standard error	*p*-value	CI lower boundary	CI upper boundary
fertilizer_type_enhanced	1.2334	0.1013	<.0001	1.0349	1.4319
fertilizer_type_combined	1.0177	0.1230	<.0001	0.7766	1.2587
fertilizer_type_organic	0.2806	0.1583	0.0764	-0.0297	0.5909
fertilizer_type_mineral	0.4063	0.1183	<.001	0.1744	0.6382
fertilizer_strategy_placement	0.9990	0.1583	<.0001	0.6887	1.3093
fertilizer_strategy_rate	0.9923	0.0998	<.0001	0.7967	1.1879
fertilizer_strategy_timing	0.6162	0.1253	<.0001	0.3707	0.8618
crop_residue_yes	0.3111	0.1154	<.01	0.0850	0.5372
cover_crop_and/or_crop_rotation	0.4683	0.2082	<.05	0.0602	0.8764
tillage:zero/reduced till	-0.4592	0.1193	<.001	-0.6929	-0.2254
crop_type_wheat	-0.0199	0.1039	0.8484	-0.2236	0.1838
crop_type_maize	0.0563	0.1181	0.6332	-0.1750	0.2877
crop_type_rice	0.0000	0.0000	0.0000	0.0000	0.0000
N_scaled	-0.1746	0.1246	0.1611	-0.4187	0.0696
Clay_scaled	-0.1196	0.0563	<.05	-0.2299	-0.0093
SOC_scaled	-0.0032	0.0638	0.9594	-0.1283	0.1218
pH_scaled	0.0579	0.0809	0.4743	-0.1007	0.2165
MAP_scaled	0.1446	0.0912	0.1127	-0.0341	0.3233
MAT_scaled	-0.1060	0.0782	0.1750	-0.2592	0.0472
N_squared_scaled	0.1757	0.1154	0.1278	-0.0504	0.4018
Placement:maize	-0.7457	0.2588	<.005	-1.2530	-0.2384
MAT_scaled:maize	0.1425	0.1149	0.2147	-0.0826	0.3676
N_scaled:SOC_scaled	-0.0208	0.0309	0.5006	-0.0814	0.0397

Scaled variables were converted to have unit variance. ROM, the log transformed ratio of means (Eq. 5); MD, the raw mean difference (Eq. 8); SMD, the standardized mean difference (Eq. 10).

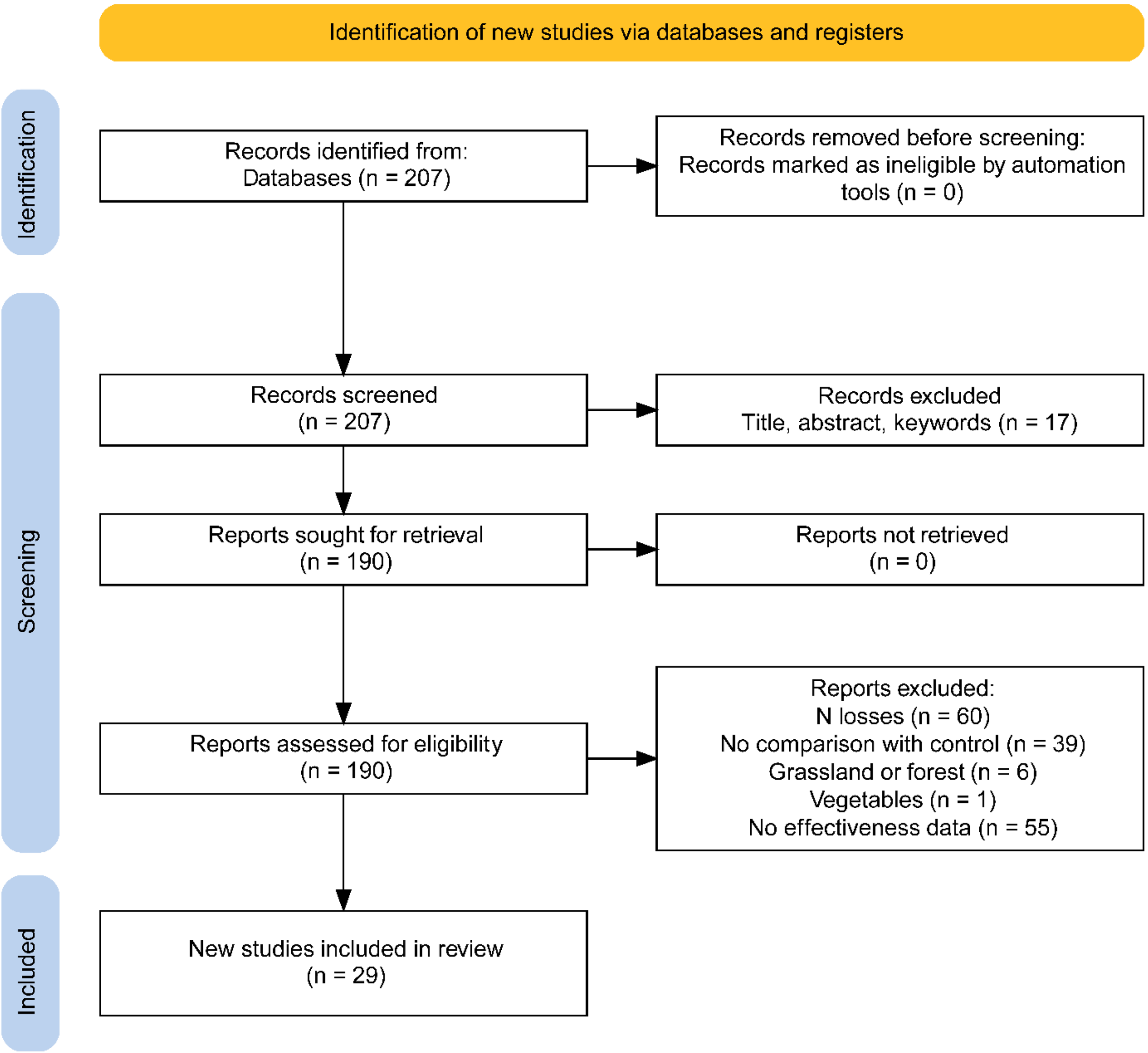

Supplementary Fig. 1 PRISMA flow diagram showing the selection process of studies included in the analysis.

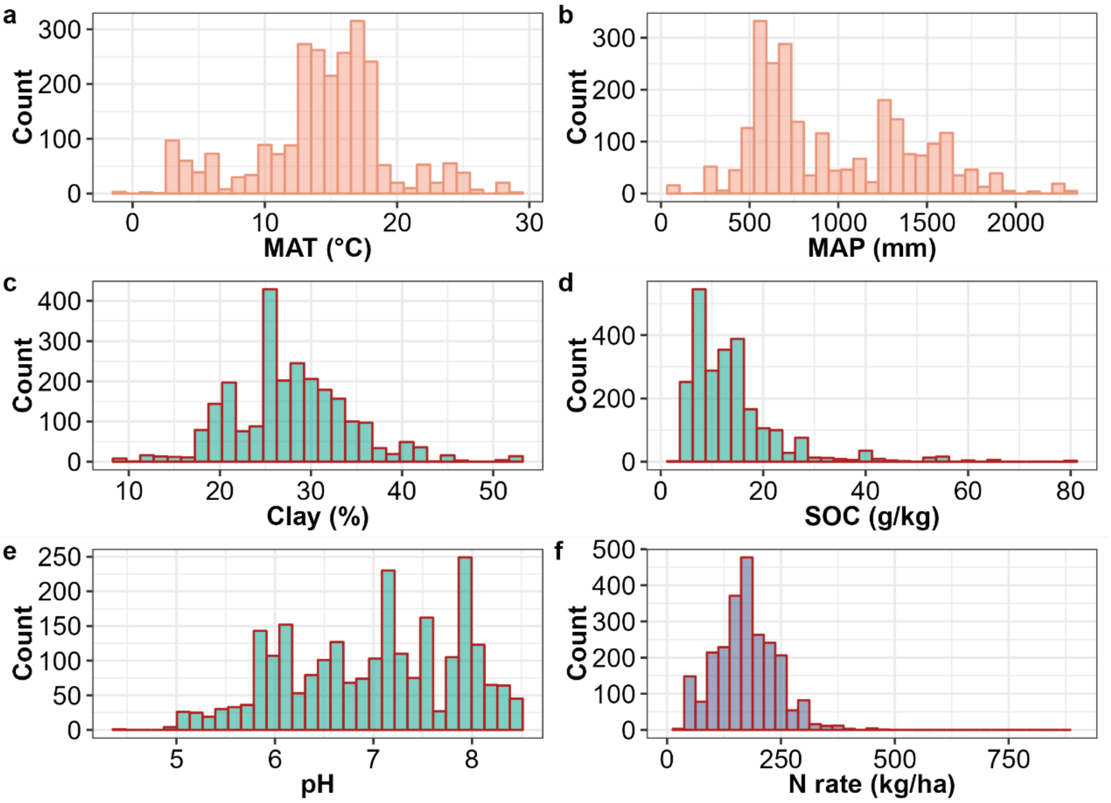

Supplementary Fig. 2 Frequency distribution histogram of MAT (a), MAP (b), Clay (c), SOC (d), pH (e), and N rate (f). MAT, mean annual temperate; MAP, mean annual precipitation; Clay, soil clay content; SOC, soil organic carbon; pH, soil pH; and N rate, N application rate. Source data are provided as a Source Data file.

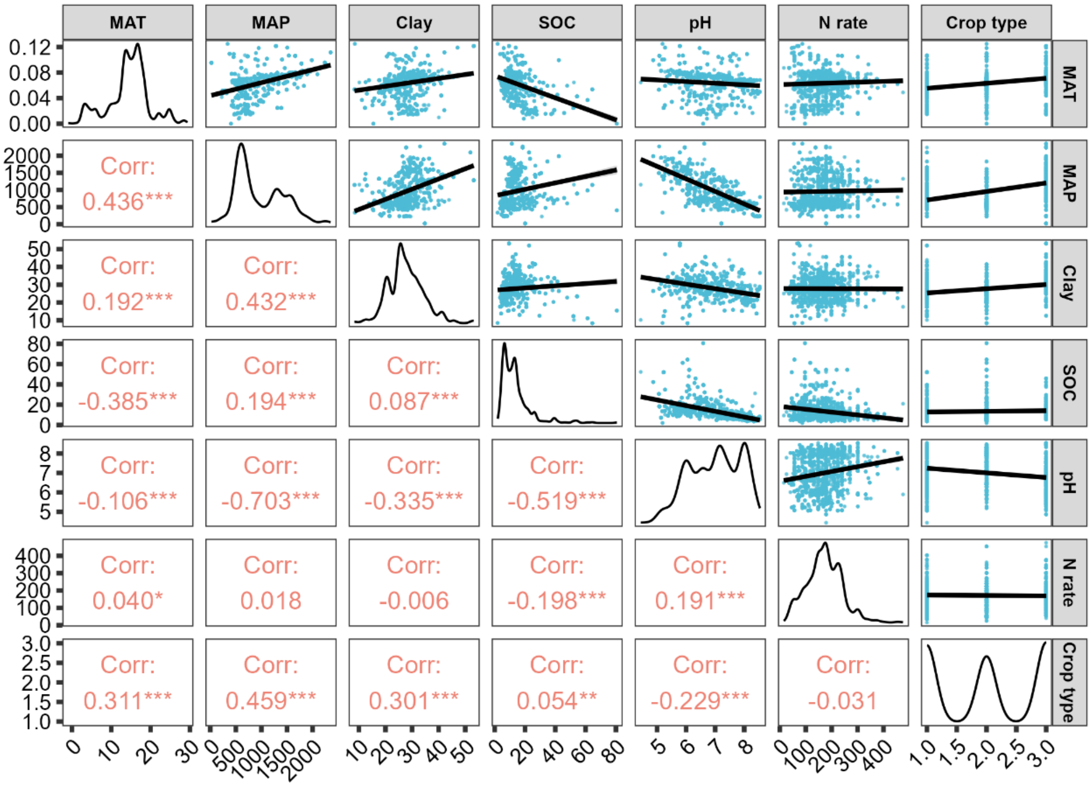

Supplementary Fig. 3 Data fitting and correlation coefficients among MAT, MAP, Clay, SOC, pH, N rate and crop type. Explanations of the variables can be found in Table S3. MAT, mean annual temperate; MAP, mean annual precipitation; Clay, soil clay content; SOC, soil organic carbon; pH, soil pH; and N rate, N application rate. Source data are provided as a Source Data file.

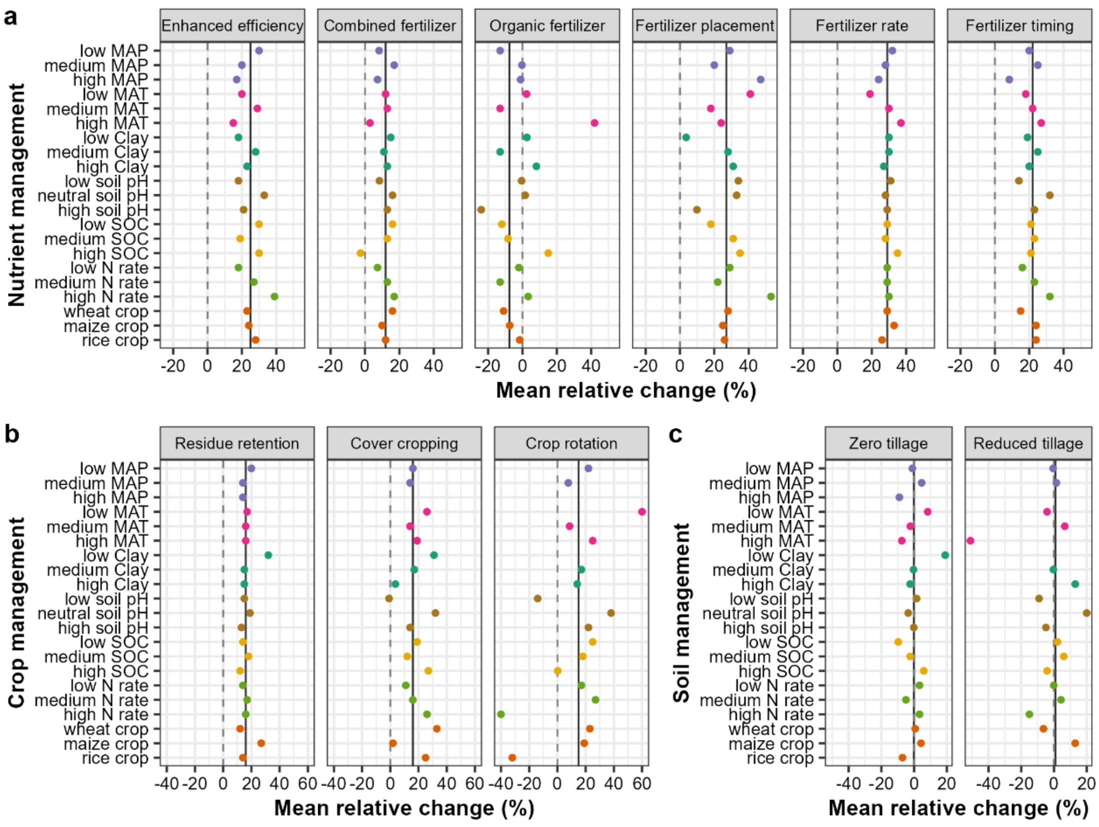

Supplementary Fig. 4 Mean effects of nutrient (a), crop (b) and soil (c) management practices, based on original source data of meta-analyses (circles), on the relative change in N recovery efficiency (%) as affected by site conditions in comparison to the overall mean impact (solid line). Dashed lines represent the zero effect, with the difference between the dashed and solid lines indicating a negative or positive effect. Means and standard deviations of effect sizes for the management practices were weighted by the number of observations. Nutrient management includes enhanced efficiency fertilizer, combined fertilizer, organic fertilizer, right fertilizer placement, right fertilizer rate and right fertilizer timing. Crop management includes residue retention, cover cropping and crop rotation. Soil management includes zero tillage and reduced tillage. Source data are provided as a Source Data file.

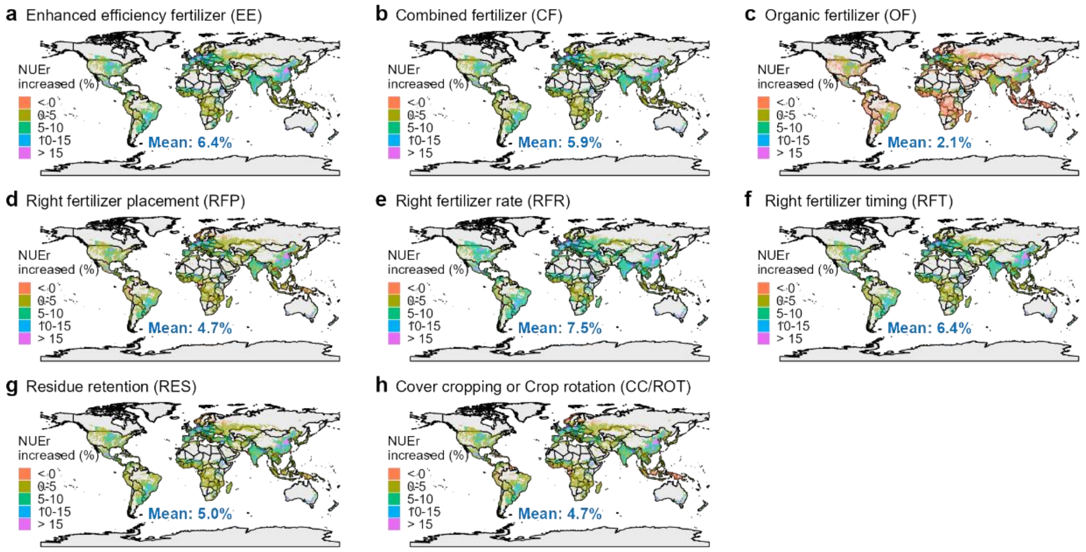

Supplementary Fig. 5 Upscaled results for global impacts of management practices on changes in absolute N recovery efficiency (%) based on the MD model. (a) EE, enhanced efficiency fertilizer; (b) CF, combined fertilizer; (c) OF, organic fertilizer; (d) RFP, Right fertilizer placement; (e) RFR, right fertilizer rate; (f) RFT, right fertilizer timing; (g) RES, residue retention; and (h) CC/ROT, cover cropping or crop rotation. Source data are provided as a Source Data file.
