# Supplementary material for: Global mean nitrogen recovery efficiency in croplands can be enhanced by optimal nutrient, crop and soil management practices
Source: Nat Commun. 2023 Sep 16;14:5747. doi: 10.1038/s41467-023-41504-2 (PMC10505174; doi:10.1038/s41467-023-41504-2)
Supplement: Supplementary file 2 — Reporting Summary [file 41467_2023_41504_MOESM2_ESM.pdf]

nature portfolio

Corresponding author(s): Yongliang Chen

Last updated by author(s): Sep 4, 2023

Reporting Summary

Nature Portfolio wishes to improve the reproducibility of the work that we publish. This form provides structure for consistency and transparency in reporting. For further information on Nature Portfolio policies, see our Editorial Policies and the Editorial Policy Checklist.

Statistics

For all statistical analyses, confirm that the following items are present in the figure legend, table legend, main text, or Methods section.

n/a	Confirmed	
☐	☒	The exact sample size (*n*) for each experimental group/condition, given as a discrete number and unit of measurement
☒	☐	A statement on whether measurements were taken from distinct samples or whether the same sample was measured repeatedly
☐	☒	The statistical test(s) used AND whether they are one- or two-sided<br>*Only common tests should be described solely by name; describe more complex techniques in the Methods section.*
☐	☒	A description of all covariates tested
☐	☒	A description of any assumptions or corrections, such as tests of normality and adjustment for multiple comparisons
☐	☒	A full description of the statistical parameters including central tendency (e.g. means) or other basic estimates (e.g. regression coefficient) AND variation (e.g. standard deviation) or associated estimates of uncertainty (e.g. confidence intervals)
☐	☒	For null hypothesis testing, the test statistic (e.g. *F*, *t*, *r*) with confidence intervals, effect sizes, degrees of freedom and *P* value noted<br>*Give P values as exact values whenever suitable.*
☒	☐	For Bayesian analysis, information on the choice of priors and Markov chain Monte Carlo settings
☐	☒	For hierarchical and complex designs, identification of the appropriate level for tests and full reporting of outcomes
☒	☐	Estimates of effect sizes (e.g. Cohen's *d*, Pearson's *r*), indicating how they were calculated

Our web collection on statistics for biologists contains articles on many of the points above.

Software and code

Policy information about availability of computer code

Data collection	Literature survey was conducted in Web of Science (https://www.webofscience.com). For the data presented graphically, the digital software GetData Graph Digitizer 2.25 (http://getdata-graph-digitizer.com) was used. Data was structured and coded using Microsoft Excel spreadsheet.
Data analysis	Data was analysed using custom code written in R version 4.2.2. Code are available on GitHub at https://github.com/gerardhros/phd_luncheng/tree/main/articles/ncoms23

For manuscripts utilizing custom algorithms or software that are central to the research but not yet described in published literature, software must be made available to editors and reviewers. We strongly encourage code deposition in a community repository (e.g. GitHub). See the Nature Portfolio guidelines for submitting code & software for further information.

Data

Policy information about availability of data

All manuscripts must include a data availability statement. This statement should provide the following information, where applicable:

- Accession codes, unique identifiers, or web links for publicly available datasets
- A description of any restrictions on data availability
- For clinical datasets or third party data, please ensure that the statement adheres to our policy

N inputs by fertilizer and manure is available from: PANGAEA (Data Publisher for Earth & Environmental Science) database (https://doi.pangaea.de/10.1594/PANGAEA.871980). Climate data is available from CRU (http://www.cru.uea.ac.uk/data). Land use data is available from the SPAM (Spatial Production Allocation

Model) dataset (https://www.mapspam.info/data). Soil property data is available from Soil Grids (http://www.isric.org/explore/soilgrids). The raw data are available on GitHub at https://github.com/gerardhros/phd_luncheng/tree/main/articles/ncoms23.

Human research participants

Policy information about studies involving human research participants and Sex and Gender in Research.

Reporting on sex and gender	NA
Population characteristics	NA
Recruitment	NA
Ethics oversight	NA

Note that full information on the approval of the study protocol must also be provided in the manuscript.

Field-specific reporting

Please select the one below that is the best fit for your research. If you are not sure, read the appropriate sections before making your selection.

☐ Life sciences ☐ Behavioural & social sciences ☒ Ecological, evolutionary & environmental sciences

For a reference copy of the document with all sections, see nature.com/documents/nr-reporting-summary-flat.pdf

Ecological, evolutionary & environmental sciences study design

All studies must disclose on these points even when the disclosure is negative.

Study description	In this study, we used meta-analytical and meta-regression models, to evaluate and predict the impacts of management practices on NUEr as a function of site factors. We first developed a meta-model by combining existing meta-analytical regression models (n = 29) to predict the change in NUEr in response to agronomic practices and its dependency on site factors. We compared these outcomes to the results of a single meta-regression model based on 2,436 paired observations from 407 primary studies. We finally evaluated the impact of management and site factors controlling NUE, and applied this model to predict the spatial variation of the potential impact of agronomic management practices on NUEr as a function of site factors at a global scale.
Research sample	For meta-analytical data, we performed a literature search for meta-analytical studies on the effect sizes for NUEr or N uptake in response to changes in nutrient, crop and soil management. Searches were performed using Web of Science (https://www.webofscience.com) with search terms: NUEr, N uptake, nutrient management, crop management, soil management and meta-analysis. This search and selection resulted in the inclusion of 29 studies. For primary data, the relevant crop, soil and nutrient management data and site-specific factors were retrieved from the 407 primary studies based on the above 29 meta-analytical studies. This resulted in 2,436 paired observations.<br>We estimated the global potential for NUEr improvements on a 0.5 x 0.5 degree resolution using existing global data sets: (1) N-inputs by fertilizer and manure from PANGAEA database (https://doi.pangaea.de/10.1594/PANGAEA.871980); (2) climate data from CRU (http://www.cru.uea.ac.uk/data); (3) land use data from the SPAM dataset (https://www.mapspam.info/data); and (4) soil properties from Soil Grids (http://www.isric.org/explore/soilgrids).
Sampling strategy	The 29 meta-analytical studies included four criteria: (1) linked to at least one management practice to the impact of NUEr or N uptake; (2) limited to management of main cereal croplands (maize, wheat and rice), excluding grasslands and forests; (3) providing estimates based on field studies, thus excluding laboratory or incubation studies; and (4) when meta-analytical studies presented a summary of previous analyses, only the most recent study was selected. For the 407 primary studies, the following variables were extracted: (1) reference details including author, title and publication year; (2) latitude and longitude; (3) experiment duration; (4) site-specific soil properties and climatic factors; (5) crop type; (6) number of replicates; (7) management practices applied (in predefined crop, soil and nutrient management classes); (8) mean NUE or N uptake in experimental and control treatments; and (9) practices of variation (including standard error, 95% confidence interval, or standard deviation).
Data collection	Luncheng You and Qi Shao conducted the literature search, screened all literature, extracted all the effect sizes, and coded the information from the studies.
Timing and spatial scale	We collected the meta-analytical data from publications before December 2021. A first literature search was conducted in January 2021. Following peer-review process, we updated our search string in Supplementary Note 1. The included studies were published between 1979 and 2021.
Data exclusions	Limited to management of croplands, excluding grasslands and forests. Providing estimates based on field studies, thus excluding laboratory or incubation studies.
Reproducibility	Our study is an integrated study mainly based on meta-model and published reference data. Our results can be reproduced when following the described methods and data.

Randomization	This is not relevant to our study because our work is not an "Experimental" study but an integrated data analysis. We used global datasets and published reference data to do the integrated analysis.
Blinding	Blinding is not possible in our study. Because we have no choice to collect the global datasets or published reference data (just according to key words) considering the blinding principle.

Did the study involve field work? ☐ Yes ☒ No

Reporting for specific materials, systems and methods

We require information from authors about some types of materials, experimental systems and methods used in many studies. Here, indicate whether each material, system or method listed is relevant to your study. If you are not sure if a list item applies to your research, read the appropriate section before selecting a response.

Materials & experimental systems

n/a	Involved in the study
☒	☐ Antibodies
☒	☐ Eukaryotic cell lines
☒	☐ Palaeontology and archaeology
☒	☐ Animals and other organisms
☒	☐ Clinical data
☒	☐ Dual use research of concern

Methods

n/a	Involved in the study
☒	☐ ChIP-seq
☒	☐ Flow cytometry
☒	☐ MRI-based neuroimaging
