# Supplementary material for: Global mean nitrogen recovery efficiency in croplands can be enhanced by optimal nutrient, crop and soil management practices
Source: Nat Commun. 2023 Sep 16;14:5747. doi: 10.1038/s41467-023-41504-2 (PMC10505174; doi:10.1038/s41467-023-41504-2)
Supplement: Supplementary file 5 — Description of Additional Supplementary Files [file 41467_2023_41504_MOESM5_ESM.pdf]

File Name: Supplementary Data 1

Description: The supplementary data 1 included 29 meta-analytical studies data. Detailed information about these studies in the database includes bibliographic details, crop types, management practices, number of observations and response variables. The management practices include nutrient management (enhanced efficiency fertilizer, combined fertilizer, organic fertilizer, mineral fertilizer, fertilizer placement, fertilizer rate and fertilizer timing), crop management (residue retention, cover cropping and crop rotation) and soil management (zero and reduced tillage).

File Name: Supplementary Data 2

Description: The Supplementary Data 2 included 407 primary studies data. The following variables are included in the database: (1) reference details including author, title and publication year; (2) latitude and longitude; (3) experiment duration; (4) site-specific soil properties and climatic conditions; (5) crop type; (6) number of replicates; (7) management practices (including nutrient, crop and soil management); (8) mean NUEr in experimental and control treatments; and (9) practices of variation.
